# Insight into the Hypoglycemic Effects of *Pinus nigra* Arn. Bark Extracts Through In Silico and In Vivo Analysis

**DOI:** 10.3390/plants15030462

**Published:** 2026-02-02

**Authors:** Nemanja Maletin, Nikola Denda, Maja Milanović, Nataša Milić, Nina Pavkov, Aleksandar Rašković, Milica Paut Kusturica

**Affiliations:** 1 Department of Pharmacology, Toxicology and Clinical Pharmacology, Faculty of Medicine Novi Sad, University of Novi Sad, 21000 Novi Sad, Serbia; 902018d24@mf.uns.ac.rs (N.D.); aleksandar.raskovic@mf.uns.ac.rs (A.R.); milica.paut-kusturica@mf.uns.ac.rs (M.P.K.); 2 Clinic of Internal Oncology, Oncology Institute of Vojvodina, 21204 Novi Sad, Serbia; 3 Clinic for Eye Diseases, University Clinical Center of Vojvodina, 21000 Novi Sad, Serbia; 4 Department of Pharmacy, Faculty of Medicine Novi Sad, University of Novi Sad, 21000 Novi Sad, Serbia; maja.milanovic@mf.uns.ac.rs (M.M.); natasa.milic@mf.uns.ac.rs (N.M.); 5 Faculty of Pharmacy Novi Sad, University Business Academy in Novi Sad, 21000 Novi Sad, Serbia; nina.bukumirovic@gmail.com

**Keywords:** *Pinus nigra* bark, plant metabolites, hypoglycaemic, hypolipidaemic, in silico, in vivo

## Abstract

Diabetes mellitus is a major global health burden, and plant-derived polyphenols are increasingly explored as adjuncts for metabolic control. Hence, the hypoglycaemic potential of *Pinus nigra* bark extract from Serbia was evaluated using complementary in silico and in vivo approaches. Major constituents reported for *P. nigra* bark (catechin, epicatechin, taxifolin, caffeic, ferulic, p-coumaric, protocatechuic, and syringic acids) were docked against selected metabolic targets (LXRα, LXRβ, PTP1B, and SUR1) as hypothesis-generating screening due to the frequent PAINS behaviour of small polyphenols. For in vivo assessment, normoglycaemic and alloxan-induced diabetic Wistar rats received a 7-day oral treatment with ethanol bark extract (100 mg/kg) alone or combined with metformin (100 mg/kg) or gliclazide (10 mg/kg), and fasting glycaemia, oral glucose tolerance, lipid profile, and body weight were assessed. The ethanol extract reduced glycaemia, improved glucose tolerance, and favourably modulated dyslipidaemia, with additive effects observed in combinations with metformin or gliclazide. These findings suggest activity relevant to hypoglycaemic-relevant activity of *P. nigra* bark extract in vivo; however, comprehensive chemical profiling, mechanistic confirmation, and safety evaluation are required before translational consideration.

## 1. Introduction

Diabetes mellitus (DM) represents one of the leading global health burdens, with a constant increase in prevalence worldwide [1]. DM is a disorder of carbohydrate, fat, and protein metabolism, primarily characterised by inadequate insulin secretion or action, leading to the inability to maintain normal blood glucose levels [2]. In addition to impaired glucose metabolism, the disease is often accompanied by dyslipidaemia and oxidative stress, which further contribute to the development of microvascular and macrovascular complications [3]. Long-term complications of diabetes, such as vascular diseases, retinopathy, and nephropathy, pose a major therapeutic challenge [2]. According to recent reports, the global prevalence of diabetes, currently affecting about 422 million people, is expected to rise to 622 million by 2040 [4,5,6].

Alongside conventional therapies for the treatment of diabetes, increasing attention has been directed toward natural substances with antioxidant, hypoglycaemic, and hypolipidaemic activities. Among these, plant extracts rich in phenolic compounds have emerged as promising candidates for metabolic modulation. One such source of bioactive metabolites is the bark of the black pine (*Pinus nigra*), which contains high concentrations of polyphenols, proanthocyanidins, and flavonoids [7]. Plant polyphenols, including flavonoids, tannins, phenolic acids, coumarins, and lignans, are secondary metabolites of diverse chemical structures and biological properties, well known for their ability to neutralise free radicals and mitigate oxidative damage at the cellular and subcellular levels [8,9].

As demonstrated by Rana et al., polyphenols exert a broad spectrum of biological effects, including antioxidant and anti-inflammatory actions, neuroprotection, and modulation of glucose and lipid metabolism, thereby contributing to the prevention of chronic non-communicable diseases such as cardiovascular and metabolic disorders [10]. Among polyphenol-rich natural sources, black pine bark extract—particularly its standardised formulation, Pycnogenol^®^—has been extensively studied and shown to possess potent antioxidant and hepatoprotective properties in experimental models, effectively reducing oxidative stress and acetaminophen-induced hepatic injury [11]. Furthermore, both experimental and clinical investigations have confirmed its favourable metabolic effects, including improved glycaemic control, lipid profile, and body weight regulation [12,13]. A recent comprehensive review of randomised, double-blind, placebo-controlled clinical trials reaffirmed these benefits, reporting consistent antioxidant, anti-inflammatory, and metabolic improvements in individuals with type 2 diabetes and metabolic syndrome [14]. Building upon these findings, our previous studies also demonstrated the pronounced antioxidant and hepatoprotective potential of Pycnogenol^®^ [11], providing a strong rationale to explore whether bark extracts derived from locally grown black pine (*Pinus nigra*) could exhibit similar pharmacological activities in experimental models.

Recent experimental evidence further supports the hypoglycaemic and hypolipidaemic potential of *Pinus* species through multiple mechanisms. Ethanolic and methanolic bark extracts of *Pinus nigra* (*P. nigra*) subsp. *laricio* demonstrated potent α-amylase and pancreatic lipase inhibition, suggesting direct modulation of carbohydrate and lipid digestion [13]. Recent studies have further documented the antioxidant potential of *P. nigra* and related pine species and linked polyphenol-rich extracts with additional bioactivities, including photoprotective effects [15]. In vitro assays confirmed that *P. nigra* bark possesses high antioxidant and radical-scavenging capacities, which may contribute to the protection of pancreatic β-cells and improved insulin sensitivity [7,11,12]. Additionally, bark extracts of *Pinus pinaster* and *Pinus brutia* were shown to reduce blood glucose, total cholesterol, and triglyceride levels in alloxan- and streptozotocin-induced diabetic models [16,17,18], indicating a cross-species metabolic benefit linked to their shared polyphenolic composition. Moreover, extracts of *Pinus densiflora* and *Pinus koraiensis* have been reported to activate AMP-activated protein kinase (AMPK), a key regulator of glucose uptake and fatty acid oxidation, thereby supporting their use in experimental models of metabolic syndrome [19].

Collectively, these findings suggest that phenolic-rich *Pinus* bark extracts exert multifactorial effects on glucose and lipid metabolism, providing a scientifically grounded rationale for testing *P. nigra* bark extract in animal models of diabetes. Unlike previous studies that primarily focused on commercial preparations, this is the first investigation to examine the therapeutic potential of *P. nigra* bark extract originating from the Republic of Serbia as a natural intervention targeting metabolic dysregulation. Accordingly, the present study aims to evaluate the hypoglycaemic, hypolipidaemic, and nutritional–metabolic effects of black pine bark through complementary in silico and in vivo approaches.

## 2. Materials and Methods

### 2.1. Plant Material and Preparation of Extract

Outer bark of black pine (*P. nigra* Arn.) was collected as a residue from the debarking process in April 2018 at Mokra Gora, Serbia, and was supplied by the Institute of Forestry, Belgrade, Serbia, where its taxonomic identity was confirmed. Bark was air-dried at room temperature in the dark, milled (3 mm sieve), and stored in paper bags in a dry, cool, dark place protected from light and moisture until further analysis. Extracts were prepared in May 2018 and analysed within 48 h after preparation (May 2018) while the in vivo experiments were conducted in June–July 2018 using freshly prepared extracts. A voucher specimen (No. 258/18) has been deposited and is publicly available in the Herbarium of the Institute of Forestry (BEOIF), Belgrade, Serbia. More details about the formal identification of the plant material and preparation of extract can be found in our previous publication [7]. For the preparation of extract, 2.5 g of powdered bark were used and extracted with 125 mL of ethanol, methanol or acetone by ultrasound-assisted extraction (40 min, ambient temperature) [20]. The filtrate was concentrated to dryness at 50 °C under reduced pressure to obtain a dry extract, stored in amber glass at room temperature (≈20 °C), and protected from light for a maximum of 48 h until use. Based on preliminary chemical profiling, the ethanol extract from Mokra Gora (richest in phenolics/proanthocyanidins) was selected for all in vivo experiments, and fresh dry ethanol extracts were always prepared following the same procedure in order to ensure good stability of phytoconstituents.

The collection of *P. nigra* bark material was conducted by the Institute of Forestry (Belgrade, Serbia) in accordance with national forestry legislation (Law on Forests, Official Gazette of the Republic of Serbia No. 30/2010, 93/2012, 89/2015, 95/2018, 91/2021) and the Law on Nature Protection (Official Gazette of the Republic of Serbia No. 36/2009, 88/2010, 91/2010 corr., 14/2016, 95/2018, 71/2021). Sampling was performed under authorization of the Ministry of Agriculture, Forestry and Water Management of the Republic of Serbia, and no protected or endangered species were collected.

### 2.2. Chemical Characterisation of the Extract

The chemical characterisation of *P. nigra* bark extracts was carried out by Milić and colleagues, determining the total phenolic content (TPC), flavonoid content (TFC), tannin content (TTC), and proanthocyanidin content (PAC) in samples from Serbia, using methanol, ethanol, and acetone as solvents [7]. The results showed a marked dependence of the chemical profile on the choice of solvent and geographical origin. The highest contents of total phenolics (35.68 mg GAE/g), flavonoids (1.22 mg QE/g), tannins (21.64 mg GAE/g), and proanthocyanidins (3.76 mg CE/g) were recorded in the ethanol extract of bark from Mokra Gora. The methanol extract from Tara contained 32.5 mg GAE/g of phenolics and 21.31 mg GAE/g of tannins, while the acetone extracts generally exhibited the lowest levels of phenolic metabolites. Based on these findings, the ethanol extract from Mokra Gora (Serbia), as the variant with the richest phenolic profile, was selected for further in vivo investigation of its hypoglycaemic and hypolipidemic potential.

### 2.3. Computational Molecular Docking Analysis

Based on the fingerprint of Pycnogenol^®^ and the preliminary chemical characterisation of the obtained *P. nigra* Arn. bark extracts [7,21], caffeic acid, catechin, epicatechin, ferulic acid, *p*-coumaric acid, protocatechuic acid, syringic acid, and taxifolin were selected for in silico analysis as representative low-molecular-weight polyphenolic markers commonly reported in pine bark preparations. Simplified Molecular Input Line Entry System (SMILES) specification of selected phytoconstituents was obtained from the PubChem database (https://pubchem.ncbi.nlm.nih.gov/). All examined metabolites have molecular mass less than 305 g/mol, hydrogen bond acceptors ≤ 7, hydrogen bond donors ≤ 5, polar surface area ≤ 128 Å^2^, and lipophilicity expressed as logP less than 1.5. All parameters were determined in silico by SwissADME (http://www.swissadme.ch/).

Chem3D (v. 16.0) software was applied for conversion of SMILES specifications into the 3D structure. In addition, the geometry optimisation of all observed metabolites was performed using Merck Molecular Force Field (MMFF94) minimisation. Molecular docking of metabolites towards selected targets related to hyperlipidaemia and diabetes was performed with Genetic Optimisation for Ligand Docking (GOLD) with Hermes module (v. 2022.3.0) on a Windows 10 operating system. The X-ray crystallographic structures of liver X receptor alpha (LXRα, PDB: 1UHL), liver X receptor beta (LXRβ, PBD: 1UPV), as well as protein tyrosine phosphatase 1B (PTP1b, PDB: 2QBP) and sulfonylurea receptor 1 (SUR1, PDB: 6PZA) were obtained from the Protein Data Bank (https://www.rcsb.org/). All structures were prepared with the Wizard tool of the GOLD software (Cambridge, United Kingdom) that allows the addition of missing hydrogen atoms and the removal of water molecules. The target binding site was set within the 6 Å of the reference ligand in order to filter out ligands with the low-affinity band. The docking protocol for each target was validated by re-docking of co-crystallised ligands (T-0901317 for LXRα and LXRβ, 5-(3-{[1-(benzylsulfonyl)piperidin-4-yl]amino}phenyl)-4-bromo-3-(carboxymethoxy)thiophene-2-carboxylic acid for PTP1b and glibenclamide for SUR1), and the obtained root mean square distance values were less than 2 Å. ChemPLP was used as a scoring function. The docking results were discussed according to the obtained highest ChemPLP Fitness score that corresponds to the best ligand-target pose [22,23,24]. Visualisation of the binding mode was performed with the academic version of Maestro Schrödinger software (v.14.1). Molecular docking was conducted as targeted docking centred on co-crystallised ligands; protocol validation employed re-docking with RMSD < 2.0 Å. Given the non-specific binding risk of small polyphenolic metabolites and their frequent PAINS flags, docking outputs are treated as hypothesis-generating only (i.e., not evidence of affinity or mechanism).

### 2.4. In Vivo Experimental Design

This study used healthy white Wistar laboratory rats of both sexes, weighing between 150 and 300 g. All animals were obtained from the accredited breeding colony of the Military Medical Academy (Belgrade, Serbia), operating under the supervision of the Veterinary Directorate of the Republic of Serbia. Animals were bred and maintained under pathogen-free conditions before transfer to the Department of Pharmacology, Toxicology and Clinical Pharmacology, Faculty of Medicine, University of Novi Sad, where the experiments were conducted. Upon arrival, animals were allowed a 7-day acclimatization period under controlled housing conditions prior to experimental procedures. During housing, the ambient temperature was maintained at 20–25 °C with controlled relative humidity and a 12 h light/12 h dark cycle. Throughout the study, animals had free access to fresh water and standard pelleted chow for small laboratory rodents (Veterinary Institute Zemun, Serbia). Food was withdrawn for six hours prior to glycaemia measurements to standardise glycoregulatory parameters.

The effects of *P. nigra* bark extract, administered alone or in combination with metformin and gliclazide, were investigated in normoglycaemic and alloxan-induced diabetic rats. Six animals were included in each experimental and control group, with each animal used only once. Body weight was monitored in all experimental animals, both normoglycaemic and alloxan-induced diabetic, to assess nutritional status and potential metabolic effects of the applied treatments. Measurements were taken using a digital scale with 0.1 g precision, immediately before and after the seven-day treatment period. Changes in body weight (∆BW) were calculated for each animal. Diabetes was induced via a single intraperitoneal injection of alloxan monohydrate at the appropriate dose. Alloxan was chosen to induce an insulin-deficient diabetic phenotype via preferential pancreatic β-cell vulnerability to oxidative stress, providing a rapid and widely used rat model for screening the hypoglycaemic effects of polyphenol-rich plant extracts. In addition, several prior studies investigating *Pinus* bark preparations have employed alloxan-induced diabetes, thereby facilitating comparability with the existing literature [25,26]. Animals were marked with picric acid solution for identification during the experiment. Metformin and gliclazide doses were calculated using Clarke’s formula, and all tested substances were dissolved or suspended in 1 mL of physiological saline to ensure uniform administration volume. Treatments were administered orally via gastric gavage, once daily at the same time, for seven consecutive days. At the end of the experimental period, two hours after the final administration and pharmacodynamic measurements, animals were anaesthetised with 25% urethane solution (5 mL/kg, intraperitoneally) and euthanised by cardiac puncture to collect blood samples for further biochemical analysis.

Wistar rats of both sexes were used (*n* = 6 per group; 3 males and 3 females). Animals were randomised by computer-generated block randomization (block size = 6) stratified by model (normoglycaemic vs. alloxan-induced). Allocation was implemented by personnel not involved in dosing or outcome assessment; cages/vials carried anonymized codes. Outcome assessors were blinded; dosing was performed by a separate operator; unblinding occurred after database lock. Pre-specified exclusion criteria were as follows: failure to achieve hyperglycaemia (blood glucose < 15 mmol/L at 48 h post-alloxan), procedural mishap (e.g., failed gavage), humane endpoints, or haemolysed/insufficient serum for lipid assays. Given the pilot design (*n* = 3 per sex per group), sex-stratified analyses were not pre-specified, and data from males and females were pooled.

Experimental groups and dosing: In each model (normoglycaemic and alloxan-induced diabetic), six groups (*n* = 6) received once-daily oral gavage for 7 consecutive days at 1 mL/kg:

• FIZ—physiological saline (1 mL/kg);
• MET—metformin (100 mg/kg);
• GLIC—gliclazide (10 mg/kg);
• PB—black pine (*P. nigra*) bark extract (100 mg/kg);
• PB + MET—black pine bark extract (100 mg/kg) + metformin (100 mg/kg);
• PB + GLIC—black pine bark extract (100 mg/kg) + gliclazide (10 mg/kg).

Each group contained six animals, with each animal used only once. This design allowed for parallel comparison of the effects of black pine bark extract, both alone and in combination with standard antidiabetic agents, in physiological and pathological models of glycoregulation.

Diabetes was induced by a single i.p. injection of alloxan (130 mg/kg) after a 6 h fast; glycaemia was measured 48 h after alloxan, and only rats with blood glucose ≥ 15 mmol/L were enrolled. After 7 treatment days, a modified two-point OGTT (0 and 30 min; glucose 3 g/kg, p.o.) was performed. Biochemical analyses were carried out in serum obtained by centrifugation of whole blood, with samples stored at −80 °C until analysis. The following parameters were determined: total cholesterol (HOL), triglycerides (TG), HDL cholesterol (HDL-C)—using enzymatic methods with commercial kits (bioMérieux, Marcy-l’Étoile, France and Randox Laboratories Ltd., Crumlin, County Antrim, United Kingdom); and LDL cholesterol (LDL-C)—calculated according to Friedewald’s formula. Analyses were conducted using an automated biochemical analyser (TECHNICON RA-XT, Randox, UK).

### 2.5. Ethics

All procedures involving experimental animals were carried out in accordance with current ethical principles and guidelines for the care and use of laboratory animals. Collection of plant material complied with institutional, national, and international guidelines and relevant Serbian legislation (Law on Forests, Official Gazette RS No. 30/2010 et seq.), with permission obtained through the Institute of Forestry (Belgrade, Serbia) acting under the Ministry of Agriculture, Forestry and Water Management. The used material was collected as a residue from the debarking process, without any additional felling or disturbance of living trees. The experimental protocol was approved by the Ethics Committee for the Protection of the Welfare of Experimental Animals of the University of Novi Sad (approval number: III-2014-02), as well as by the Ministry of Agriculture and Environmental Protection—Veterinary Directorate of the Republic of Serbia (decision number: 323-07-00550/2015-05).

### 2.6. Statistical Analysis

The results are presented as the mean value ± standard deviation ($\bar{x}$ ± SD). Statistical analysis was performed using IBM SPSS software package v26.0 (IBM Corp., Armonk, NY, USA). The normality of data distribution was assessed using the Shapiro–Wilk test. Differences in arithmetic means between multiple independent groups were evaluated by one-way analysis of variance (ANOVA), and Tukey’s HSD test was applied for post hoc comparisons. Differences between initial and final values within the same group were assessed using Student’s paired-sample *t*-test. Comparisons of changes between groups were performed using ANOVA on differential values (∆). A *p*-value of <0.05 was considered statistically significant.

## 3. Results

### 3.1. Results of Computational Molecular Docking Analysis

Docking simulation was applied in order to evaluate binding affinity of tested metabolites towards therapeutic targets involved in complex cell-signalling pathways related to diabetes and hyperlipidaemia. The selected PDBs of LXRα, LXRβ, PTP1b, and SUR1 were carefully chosen based on the activity of their co-crystallised ligands and the completeness of the amino acid structure. The obtained highest ChemPLP Fitness score corresponded to the best docking poses of each metabolite (Table 1).

**Figure 1:**
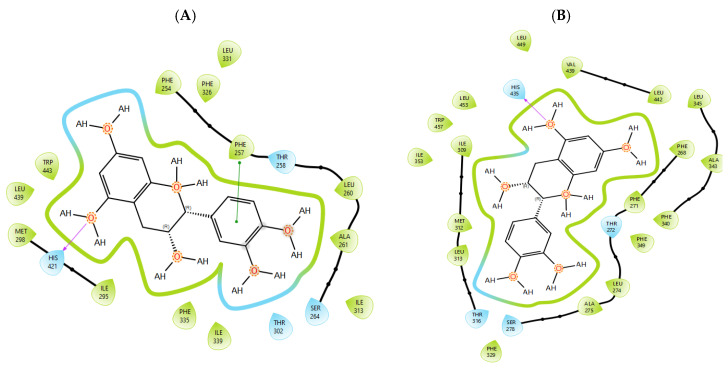
Epicatechin inside the binding pocket of (**A**) LXRα and (**B**) LXRβ. Green line was used to mark π-π interaction, while violet arrow was used for hydrogen bond.

**Figure 2:**
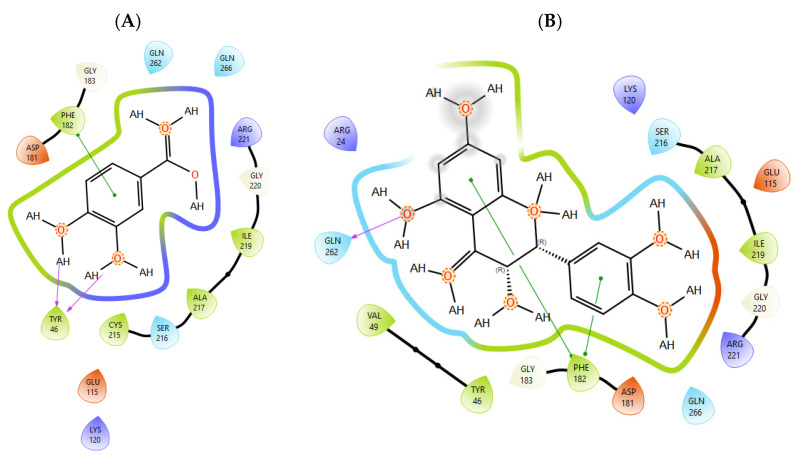
The interactions between PTP1b and (**A**) protocatechuic acid and (**B**) taxifolin. Green line was used to mark π-π interaction while violet arrow was used for hydrogen bond.

**Figure 3:**
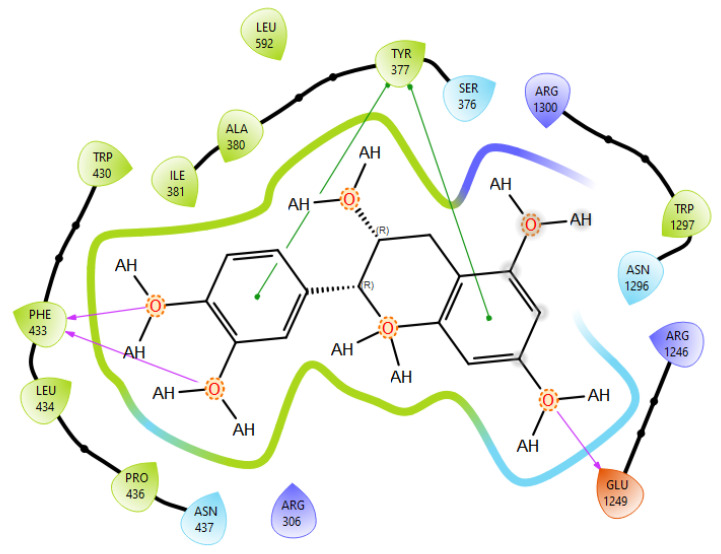
Epicatechin best docking pose at SUR1 binding site. Green line was used to mark π-π interaction while violet arrow was used for hydrogen bond.

T0901317, acting as synthetic agonist and co-crystallised ligand for LXRα and LXRβ, exhibited ChemPLP Fitness scores of 77.3841 and 83.9228, respectively. Among analysed metabolites, catechin, epicatechin, and taxifolin showed the highest scores for LXRα and LXRβ, reproducing the interaction observed for co-crystallised ligand T0901317 (Figure 1). The highest affinity towards PTP1b expressed as a ChemPLP Fitness score had caffeic acid, epicatechin, catechin, ferulic acid, and taxifolin in comparison with the proven co-crystallised ligand (ChemPLP Fitness score 101.0303). However, only protocatechuic acid made hydrogen bond with the Tyr46-like co-crystalised ligand (Figure 2). Binding affinity of selected *P. nigra*-originated metabolites towards SUR1 was evaluated in contrast to the co-crystallised ligand glibenclamide (ChemPLP Fitness Score 76.0473). Again, catechin, epicatechin, and taxifolin exhibited the highest affinity. It is worth noting that all tested metabolites inside the ligand binding domain exhibited π-π interaction with Tyr377 like glibenclamide (Figure 3).

### 3.2. Hypoglycaemic Effect of P. nigra

Normoglycaemic rats: Baseline blood glucose did not differ between groups. After 7 days of treatment, between-group differences were observed, with the lowest final glucose values recorded in the MET and PB + GLIC groups (Table 2). Within-group comparisons showed a significant decrease vs. baseline in all treated groups, whereas the magnitude of change (ΔBG) was smallest in FIZ and PB and most pronounced in MET and PB + GLIC (Table 2).

**Alloxan-induced diabetic rats:** Baseline glucose values (BG before) were comparable across groups. Two days after alloxan (BG 0), glucose differed between groups, with lower values in PB-based regimens (PB, PB + MET, PB + GLIC) compared with controls and standard-drug-only groups (Table 2). After 7 days of treatment, PB, PB + MET, and PB + GLIC achieved markedly lower final glycaemia and larger reductions (ΔBG), with the combination groups showing the strongest effect (Table 2).

To improve readability, omnibus one-way ANOVA results (F- and *p*-values) are summarised in Table S1. Baseline blood glucose did not differ significantly between groups in either experiment, whereas post-treatment measurements showed significant between-group differences in blood glucose and its change (ΔBG), most prominently in the alloxan-induced diabetic model. In addition, significant between-group differences were observed after OGTT in normoglycaemic animals.

Following the oral glucose tolerance test, post-load blood glucose levels differed significantly among groups, with the highest values observed in the FIZ group and the lowest in the PB + GLIC group (Table 3).

### 3.3. Hypolipidaemic Effect of P. nigra

In the assessment of the effects of different treatments on the lipid profile of normoglycaemic and alloxan-induced diabetic rats, significant changes were observed in the concentrations of total cholesterol, triglycerides, HDL cholesterol, and LDL cholesterol (Table 4). In normoglycaemic rats, the concentration of total cholesterol was significantly higher in the groups receiving black pine bark extract (PB), as well as in combination with metformin (PB + MET) and gliclazide (PB + GLIC), compared with the FIZ, MET, and GLIC groups (*p* < 0.05). The highest value was recorded in the PB + MET group. Conversely, triglyceride concentration was highest in the FIZ and PB + MET groups and significantly lower in the MET, GLIC, and PB groups (*p* < 0.05). The lowest triglyceride value was recorded in the MET group. HDL cholesterol concentration was statistically lowest in the FIZ group, while all groups receiving PB, either alone or in combination, showed significantly higher HDL values (*p* < 0.0001), with PB + MET and PB + GLIC standing out in particular. For LDL cholesterol, the highest values were observed in the PB, PB + MET, and PB + GLIC groups, which were significantly higher compared with the MET, FIZ, and GLIC groups (*p* < 0.05). In alloxan-induced diabetic rats, no statistically significant difference was observed in total cholesterol between groups (*p* = 0.906), but marked differences were found in other parameters. Triglyceride concentration was highest in the FIZ group and significantly higher compared with all other treatments (*p* < 0.0001). Similarly to healthy rats, HDL cholesterol was lowest in the FIZ group and significantly higher in the PB, PB + MET, and PB + GLIC groups (*p* < 0.0001). LDL cholesterol values were highest in the FIZ and MET groups, while PB treatment resulted in a significant reduction compared with the other groups (*p* < 0.0001)

### 3.4. Nutritional–Metabolic Effect of P. nigra

In assessing the impact of different treatments on rat body weight, changes were analysed in both normoglycaemic and alloxan-induced diabetic animals (Table 5). In normoglycaemic rats, paired-sample Student’s *t*-test revealed a statistically significant increase in body weight after the seven-day treatment in all experimental groups (*p* < 0.05). The greatest weight gain was recorded in the FIZ (40.0 ± 7.9 g) and PB + GLIC (38.8 ± 9.5 g) groups, while the smallest increase was observed in the MET group (19.2 ± 7.6 g), which was significantly lower compared with the PB + GLIC group (*p* < 0.05). One-way ANOVA showed significant differences in body weight change between groups (F = 7.589; *p* < 0.0001), with the MET group having significantly lower weight gain than PB + GLIC, while PB + GLIC showed significantly higher gain compared with several other groups. In diabetic rats, a statistically significant increase in body weight was observed in most groups after the seven-day treatment (*p* < 0.05), except for the PB + MET group, where a weight loss was recorded (−6.3 ± 19.5 g), though this was not statistically significant (*p* = 0.463). The highest weight gain was seen in the GLIC group (76.5 ± 14.4 g), whereas changes in PB, PB + MET, and PB + GLIC were significantly lower than in GLIC (*p* < 0.05). One-way ANOVA indicated statistically significant differences in ∆BW between the diabetic rat groups (F = 15.383; *p* < 0.0001), with the PB + MET group showing a significantly smaller weight gain compared with FIZ, MET, and GLIC groups (*p* < 0.05).

## 4. Discussion

The results of our study indicate that black pine (*P. nigra*) bark extract, particularly that originating from the mountainous regions of Serbia, possesses pronounced hypoglycaemic and hypolipidaemic potential. Its administration led to a significant reduction in glycaemia, improved glucose tolerance, and normalisation of key lipid profile parameters, suggesting its possible application in the prevention and treatment of metabolic disorders.

Given the small size and polyphenolic nature of the profiled metabolites (e.g., catechin, epicatechin, taxifolin), non-specific docking and PAINS-type behaviours are likely; therefore, in silico poses are hypothesis-generating and do not constitute evidence of target affinity or mechanism. Binding or enzymatic assays are required to substantiate these leads. Docking simulation studies enabled an insight into the binding affinities and modes of interaction between *P. nigra* bark extract-originated metabolites and targets involved in the onset and progression of diabetes. LXRs are a special type of nuclear receptors with a recognised role in cholesterol metabolism and lipid biosynthesis. Induced by insulin, LXRα and LXRβ regulate glucose production in liver and act as sensors of endoplasmic reticulum stress and inflammation [27,28]. Although all tested metabolites had lower scores in comparison with co-crystallised ligand T0901317, catechin, epicatechin, ferulic acid, protocatechuic acid, syringic acid, and taxifolin exhibited a hydrogen bond with His421 of LXRα, which is the most important for LXRα activation [29]. Moreover, ferulic acid and protocatechuic acid interacted with the target via additional hydrogen bond with Thr302, while epicatechin as T0901317 formed a π-π interaction with Phe257 (Figure 1A). A similar binding mode was observed for onion polyphenols, implying that additional bonds improve ligand stabilisation inside the LXRα binding domain [30]. On the other hand, epicatechin (Figure 1B) and taxifolin made a hydrogen bond with His435 inside the LXRβ binding pocket, reproducing the interaction observed for co-crystallised ligand T0901317. In addition, analysed metabolites had the highest affinity towards PTP1b expressed as ChemPLP Fitness score (Table 2). PTP1b, as a highly specific enzyme, is involved in insulin and leptin signalling pathways and is considered a novel therapeutic target for the management of insulin resistances, obesity, type 2 diabetes, dyslipidaemia, fatty liver, etc. [31]. Tyr46, Gln262, and Phe182 are recognised as important active site residues for inhibitory properties of a co-crystallised ligand and some phytochemicals present in plants [31,32]. Protocatechuic acid made a dense network of interaction via two hydrogen bonds with Tyr46 and π-π interaction, with Phe182 implying potent inhibition properties despite the lower ChemPLP Fitness score (48.0266, Figure 2A). Catechin, epicatechin, and taxifolin (Figure 2B) also generated a network of interactions with the surrounding amino acid residues (hydrogen bond with Gln262 and two π-π interactions with Phe182). The binding affinity towards SUR1, the regulatory subunit of the ATP-sensitive potassium (KATP) channel predominantly expressed in pancreatic β-cells, was also evaluated in silico to explore the potential insulin secretagogue-like effects of the studied phytometabolites [33]. All analysed metabolites reproduced π-π interaction with Tyr377 as glibenclamide, which is important for SUR1 activation [29,30]. The stability of epicatechin and protocatechuic acid inside the SUR1 binding site was increased through the additional interaction with the same amino residue via π-π or hydrogen bond, respectively (Figure 3). Flavanols such as fisetin, isorhamnetin, myricetin, and quercetin replicated a similar binding mode, forming a hydrophobic bond with Tyr377 [34]. The formation of interaction with Glu1249 promoted the stability of epicatechin and a p-coumaric acid receptor–ligand complex [35]. Bearing in mind that mentioned above, chemically different metabolites present in *P. nigra* Arn. bark extracts possess synergistic activity towards tested targets and might be used as an ideal combination of molecules in a multi-target approach to diabetes and hyperlipidaemia. Hence, the integration of in silico results with in vivo experiments ensured comprehensive investigation of *P. nigra* Arn. bark antidiabetic and hypolipidemic effects.

The bark extract of *P. nigra*, particularly that obtained via ethanol extraction from Mokra Gora, exhibits a marked antidiabetic effect. In our study, this extract led to a statistically significant reduction in glycaemia in diabetic animals compared with the control group and the additional administration of metformin or gliclazide in combination with the extract enhanced this effect, suggesting possible additive effects between plant polyphenols and standard antidiabetic drugs through complementary mechanisms, including improvement of insulin signalling, inhibition of digestive enzymes, stimulation of glucose uptake in peripheral tissues, and protection of pancreatic β-cells from oxidative damage [4,7,12,36,37,38,39,40].

Numerous studies indicate that various pine species from the family Pinaceae possess pronounced antidiabetic potential, attributed to phenolic metabolites, flavonoids, and catechins [16,17,18]. Although the French maritime pine (*Pinus pinaster*) has been the most extensively studied, significant levels of bioactive metabolites have also been identified in the bark of *P. nigra* and *Pinus brutia*, which contain higher levels of total phenolics and taxifolin compared with other pine species, making them potentially valuable sources of natural antioxidants [41]. Detailed compositional analyses of *P. nigra* bark extracts in the literature (including UAE/MAE-derived extracts) have shown substantial variation in polyphenolic composition and bioactivity across species and extraction methods, underscoring the need for rigorous chemical profiling and batch standardisation [42].

Several recent studies have confirmed that *P. nigra* extracts possess beneficial antidiabetic properties [12]. Inhibition of digestive enzymes responsible for carbohydrate and lipid breakdown is one of the mechanisms underlying this effect, with ethanol extracts of *Pinus nigra* subsp. *laricio* shown to effectively inhibit α-amylase and pancreatic lipase activity [13]. In addition to its glycoregulatory effects, *P. nigra* extract in our study demonstrated the ability to prevent postprandial hyperglycaemia following OGTT, further supporting its potential application in controlling glycaemic fluctuations. The effect of the ethanol extract was comparable to that of metformin, while its combination with metformin or gliclazide produced an even stronger inhibition of glycaemic rise, reinforcing the hypothesis of complementary mechanisms of action. By inhibiting key digestive enzymes, lipases, and amylases, *P. nigra* reduces the absorption of fats and carbohydrates. Lipase inhibition decreases triglyceride breakdown and caloric intake, whereas amylase inhibition slows starch digestion, thereby lowering postprandial glycaemia. The strongest α-amylase inhibitory activity was observed in the ethyl acetate fraction of apical shoot extract (IC_50_ = 22.05 ± 0.29 µg/mL), while lipase inhibition reached 0.62 mg/mL, comparable to the effect of the reference drug acarbose [13]. Similarly, Zulfqar et al. reported that methanol and ethyl acetate extracts of pine nuts (*Pinus* spp.) significantly reduced glycaemia in alloxan-induced diabetic mice after 14 days of treatment, with the most pronounced effect at a dose of 750 mg/kg [4]. The extracts also inhibited glycaemic rise during OGTT, improved lipid profile, and favourably influenced markers of liver and kidney function. According to the authors, these effects can be attributed to flavonoids and phenolic metabolites, acting through potential insulin-mimetic and antioxidant mechanisms. They also highlighted the prevention of postprandial hyperglycaemia, which is particularly important in the context of early type 2 diabetes.

In our study, *P. nigra* bark extract demonstrated a pronounced hypolipidemic effect, particularly in diabetic rats, where a significant increase in HDL cholesterol and a reduction in triglycerides and LDL cholesterol was observed compared with the control and metformin groups. Of particular note was the effect of the extract in combination with metformin, which led to triglyceride stabilisation and a marked decrease in LDL cholesterol, suggesting a synergistic mechanism of action. These findings are consistent with literature reports indicating that proanthocyanidins may influence the activity of enzymes involved in lipid metabolism and the modulation of lipoprotein profiles [43]. The study by Zulfqar et al. also demonstrated that pine extracts significantly reduce total cholesterol, triglycerides, and LDL cholesterol, while simultaneously increasing HDL cholesterol compared with diabetic controls [4]. Flavonoids have been shown to substantially lower triglyceride levels, thereby improving the lipid profile, a finding confirmed by several studies [44,45]. The hypolipidaemic effects of flavonoids are most likely mediated by inhibition of HMG-CoA reductase and pancreatic lipase, as well as activation of AMPK and modulation of lipid metabolism at the gene-expression level [13,18,46,47]. Taken together, these results suggest that *P. nigra* may have additional value in the prevention of dyslipidaemia and cardiovascular complications in diabetes, reinforcing its potential as a metabolically active phytotherapeutic agent.

In addition to the effects described above, *P. nigra* extract also influences body weight, which may be explained by its impact on glucose and lipid metabolism, as well as by its pronounced antioxidant activity. Together, these actions contribute to reducing metabolic stress and regulating body weight. The observed link between antioxidant activity and lipid metabolism regulation suggests that polyphenols, by neutralising oxidative stress, may indirectly affect the expression and activity of enzymes involved in lipid homeostasis, thereby contributing to body weight control and the prevention of metabolic disorders [48,49]. In our study, black pine bark extract showed a moderate effect on stabilising body weight in diabetic rats, with the combination of extract and metformin (PB + MET) even resulting in a slight reduction in weight. This finding is particularly important given that obesity is one of the key factors in the pathogenesis of type 2 diabetes, and the ability of the extract to limit weight gain may represent an additional advantage in the context of metabolic control and its overall antidiabetic effect. Several studies have demonstrated that extracts of different pine species, including *P. nigra*, may exert anti-obesity and hypolipidaemic effects, partly mediated by activation of the AMPK pathway, leading to reduced lipogenesis and improved oxidative status [13,18,50,51,52]. Since oxidative stress plays a crucial role in the development of insulin resistance and fatty liver disease, the use of extracts with combined antioxidant and hypolipidaemic effects could provide a valuable approach for preventing obesity-related metabolic complications [53,54]. Considering that obesity is a major comorbidity in type 2 diabetes, the capacity of *P. nigra* bark extract to stabilise or even reduce body weight represents an added therapeutic advantage in the management of metabolic syndrome.

Although the results obtained provide important insight into the hypoglycaemic and hypolipidaemic potential of *P. nigra* bark extract, several limitations should be considered when interpreting the findings. Firstly, this study did not include toxicity assessment or histopathological evaluation, which limits the appraisal of extract safety. Secondly, although the experimental design employed validated models of diabetes and the use of standard drugs, the number of experimental animals was relatively small, which may affect the strength of statistical conclusions. Moreover, this study did not involve the monitoring of inflammatory and oxidative biomarkers, nor the expression of genes involved in glucose and lipid metabolism, thereby lacking mechanistic confirmation of the observed effects. An important limitation of the present study is that a comprehensive qualitative and quantitative chemical profiling of individual constituents in the *P. nigra* bark extract was not performed within this work. Although total phenolic, flavonoid, tannin, and proanthocyanidin contents were previously reported, the absence of detailed compositional analysis (e.g., HPLC–MS-based identification and quantification) precludes direct attribution of the observed biological effects to specific compounds. Consequently, the in silico findings should be interpreted as hypothesis-generating, and future studies should include rigorous chemical characterisation alongside mechanistic validation.

## 5. Conclusions

This study provides novel in vivo evidence that a polyphenol-rich *P. nigra* bark extract from the Republic of Serbia exerts hypoglycaemic and hypolipidaemic effects in an alloxan-induced diabetic rat model, with additive efficacy when combined with metformin or gliclazide. These effects are discussed in the context of key polyphenolic markers (caffeic acid, catechin, epicatechin, ferulic acid, *p*-coumaric acid, protocatechuic acid, syringic acid, and taxifolin), and the in silico findings further support its potential as an adjunctive metabolic modulator. Further chemical profiling and mechanistic and safety validation, including dose optimisation and long-term evaluation, are required before considering nutraceutical development.

## Figures and Tables

**Table 1:** The binding affinity of analysed metabolites expressed as ChemPLP Fitness score towards LXRα (1UHL), LXRβ (1UPV), PTP1b (2QBP), and SUR1 (6PZA).

Metabolites	LXRα	LXRβ	PTP1b	SUR1
caffeic acid	42.0860	42.2480	56.5566	43.4452
catechin	53.4612	51.5348	54.7850	50.9187
epicatechin	55.8988	50.5308	56.2069	49.3273
ferulic acid	40.8389	43.5745	55.1714	44.0073
p-coumaric acid	40.6567	42.6382	53.5048	38.9443
protocatechuic acid	34.3643	39.8324	48.0266	38.2315
syringic acid	35.5140	37.0144	46.1117	37.2440
taxifolin	55.6870	48.9686	54.9619	49.9858

**Table 2:** Effect of different treatments on blood glucose levels in normoglycaemic and alloxan-induced diabetic rats.

	Normoglycaemic Rats, $\bar{x}$ ± SD (mmol/L)			Rats with Alloxan-Induced Diabetes, $\bar{x}$ ± SD (mmol/L)			
Group	BG Before	BG After	∆BG	BG Before	BG 0	BG Final	∆BG
FIZ	7.60 ± 1.29	7.25 ± 0.33 *^a^	−0.35 ± 1.26 ^b^	6.76 ± 0.30	32.5 ± 0.2 ^a^	33.0 ± 0.3 ^a^	0.5 ± 0.3 ^a^
MET	7.98 ± 0.56	5.87 ± 0.78 *^b^	−2.15 ± 0.62 ^b^	6.77 ± 0.35	34.0 ± 2.1 ^a^	29.7 ± 5.5 ^a^	−3.3 ± 5.5 ^a^
GLIC	7.72 ± 0.82	6.67 ± 0.75 *^ab^	−1.05 ± 0.59 ^ab^	6.73 ± 0.27	33.5 ± 2.9 ^a^	31.4 ± 2.4 ^a^	−2.1 ± 2.4 ^a^
PB	7.02 ± 0.32	6.43 ± 0.41 *^ab^	−0.58 ± 0.42 ^a^	6.75 ± 0.31	28.3 ± 4.4 ^b^	11.8 ± 10.5 *^b^	−16.5 ± 9.0 ^b^
PB + MET	7.05 ± 0.27	5.75 ± 0.39 *^b^	−1.30 ± 0.61 ^ab^	6.73 ± 0.34	23.6 ± 1.3 ^c^	4.6 ± 1.6 *^c^	−20.4 ± 4.8 ^c^
PB + GLIC	7.30 ± 0.74	5.25 ± 0.53 *^b^	−2.05 ± 0.94 ^b^	6.77 ± 0.26	25.6 ± 3.2 ^bc^	5.2 ± 1.^9^ *^c^	−19.1 ± 2.4 ^c^

Note: Values are mean ± SD. Different lowercase letters within the same column indicate significant differences between groups (one-way ANOVA with Tukey’s HSD, *p* < 0.05). * indicates a significant difference vs. baseline within the same group (paired-sample *t*-test; for normoglycaemic rats: BG after vs. BG before; for diabetic rats: BG final vs. BG 0), *p* < 0.05.

**Table 3:** Change in blood glucose level before and after oral glucose tolerance test (OGTT) in normoglycaemic rats.

	Normoglycaemic Rats, $\bar{x}$ ± SD (mmol/L)	
Group	BG Before OGTT	BG After OGTT
FIZ	7.60 ± 1.29	8.17 ± 0.41 ^a^
MET	7.98 ± 0.56	6.70 ± 0.48 ^b^
GLIC	7.72 ± 0.82	6.03 ± 0.64 ^b^
PB	7.02 ± 0.32	6.68 ± 0.68 ^b^
PB + MET	7.05 ± 0.27	6.57 ± 0.60 ^b^
PB + GLIC	7.30 ± 0.74	5.83 ± 0.38 ^b^

Note: Values are mean ± SD. Different lowercase letters within the same column indicate significant differences between groups (one-way ANOVA with Tukey’s HSD, *p* < 0.05).

**Table 4:** Changes in lipid levels in normoglycaemic and alloxan-induced diabetic rats.

	**Total Cholesterol (HOL), $\bar{x}$ ± SD (mmol/L)**			**Triglycerides (TG), $\bar{x}$ ± SD (mmol/L)**		
**Group**	**HOL** **Normoglycaemic**	**HOL Alloxan-** **Induced Diabetic**	**∆HOL**	**TG** **Normoglycaemic**	**TG Alloxan-** **Induced Diabetic**	**∆TG**
FIZ	1.595 ± 0.118	2.108 ± 0.395	0.513 ± 0.412	0.663 ± 0.095 ^a^	1.865 ± 0.616 ^a^	1.202 ± 0.623
PB	2.070 ± 0.096 ^a^	1.978 ± 0.473	−0.092 ± 0.483	0.477 ± 0.101 ^b^	0.475 ± 0.099 ^b^	−0.002 ± 0.141
MET	1.457 ± 0.083	2.085 ± 0.222	0.628 ± 0.237	0.372 ± 0.058 ^b^	0.868 ± 0.218 ^c^	0.496 ± 0.226
GLIC	1.650 ± 0.139	1.968 ± 0.297	0.318 ± 0.328	0.430 ± 0.152 ^b^	0.675 ± 0.148 ^c^	0.245 ± 0.212
PB + MET	2.343 ± 0.269 ^a^	2.103 ± 0.187	−0.24 ± 0.328	0.623 ± 0.119 ^a^	0.500 ± 0.113 ^b^	−0.123 ± 0.164
PB + GLIC	2.220 ± 0.081 ^a^	2.167 ± 0.078	−0.053 ± 0.112	0.488 ± 0.091 ^b^	0.427 ± 0.062 ^b^	−0.061 ± 0.11
	**HDL Cholesterol (HDL-C), $\bar{x}$ ± SD (mmol/L)**			**LDL Cholesterol (LDL-C), $\bar{x}$ ± SD (mmol/L)**		
**Group**	**HDL** **Normoglycaemic**	**HDL Alloxan-** **Induced Diabetic**	**∆HDL**	**LDL** **Normoglycaemic**	**LDL Alloxan-** **Induced Diabetic**	**∆LDL**
FIZ	0.507 ± 0.138 ^a^	0.582 ± 0.088 ^a^	0.075 ± 0.164	0.762 ± 0.051 ^a^	0.952 ± 0.024 ^a^	0.19 ± 0.056
PB	1.053 ± 0.112 ^a^	1.142 ± 0.153 ^a^	0.089 ± 0.19	0.800 ± 0.042 ^a^	0.800 ± 0.097	0.0 ± 0.106
MET	0.700 ± 0.067 ^b^	0.770 ± 0.124 ^b^	0.07 ± 0.141	0.643 ± 0.061 ^b^	0.957 ± 0.090	0.314 ± 0.109
GLIC	0.802 ± 0.056 ^a^	0.700 ± 0.141 ^a^	−0.102 ± 0.152	0.728 ± 0.051 ^a^	0.912 ± 0.048	0.184 ± 0.07
PB + MET	1.195 ± 0.150 ^b^	1.123 ± 0.083 ^b^	−0.072 ± 0.171	0.865 ± 0.083 ^a^	0.865 ± 0.057	0.0 ± 0.101
PB +GLIC	1.200 ± 0.060 ^b^	1.060 ± 0.072 ^b^	−0.14 ± 0.094	0.800 ± 0.023 ^a^	0.848 ± 0.021	0.048 ± 0.031

Note: Values are mean ± SD. Δ values represent the difference between the alloxan-induced diabetic and normoglycaemic conditions within the same treatment group (Δ = diabetic − normoglycaemic). Different lowercase letters within the same column indicate significant differences between groups (one-way ANOVA with Tukey’s HSD, *p* < 0.05).

**Table 5:** Changes in body weight (BW [g]) in normoglycaemic and alloxan-induced diabetic rats.

	Normoglycaemic Rats, $\bar{x}$ ± SD (g)			Rats with Alloxan-Induced Diabetes, $\bar{x}$ ± SD (g)		
Group	BW Start	BW Final	∆BW	BW Start	BW Final	∆BW
FIZ	276.8 ± 16.5	316.8 ± 17.4 *^a^	40.0 ± 7.9 ^a^	183.8 ± 9.0	201.5 ± 8.2 *^a^	17.7 ± 8.7 ^a^
MET	280.5 ± 11.0	299.7 ± 16.6 *^b^	19.2 ± 7.6 ^b^	166.3 ± 16.8	199.0 ± 11.0 *^a^	32.7 ± 14.5 ^b^
PB	215.7 ± 16.1	251.5 ± 18.7 *^c^	35.8 ± 5.1 ^a^	261.33 ± 5.7	262.0 ± 15.2 *^b^	0.7 ± 16.1 ^c^
GLIC	250.0 ± 12.1	273.2 ± 22.1 *^bc^	23.2 ± 10.2 ^b^	178.0 ± 14.2	254.5 ± 26.2 *^c^	76.5 ± 14.4 ^d^
PB + MET	218.5 ± 14.4	273.8 ± 15.8 *^bc^	24.3 ± 10.3 ^c^	271.5 ± 13.0	265.2 ± 23.0 *^b^	−6.3 ± 19.5 ^c^
PB + GLIC	213.7 ± 9.6	256.7 ± 17.5 *^c^	38.8 ± 9.5 ^a^	258.7 ± 13.7	267.3 ± 14.0 *^b^	8.7 ± 12.4 ^c^

Note: Values are mean ± SD. Different lowercase letters within the same column indicate significant differences between groups (one-way ANOVA with Tukey’s HSD, *p* < 0.05). * indicates a significant difference vs. BW start within the same group (paired-sample *t*-test, *p* < 0.05).

## Data Availability

The original contributions presented in this study are included in the article/Supplementary Material. Further inquiries can be directed to the corresponding author.

## Supplementary Materials

The following supporting information can be downloaded at: https://www.mdpi.com/article/10.3390/plants15030462/s1, Table S1: Summary of one-way ANOVA results for blood glucose and OGTT outcomes.
