# Supplementary material for: Insight into the Hypoglycemic Effects of Pinus nigra Arn. Bark Extracts Through In Silico and In Vivo Analysis
Source: Plants (Basel). 2026 Feb 2;15(3):462. doi: 10.3390/plants15030462 (PMC12899274; doi:10.3390/plants15030462)
Supplement: Supplementary file 1 [file plants-15-00462-s001.zip › plants-4071891-supplementary.pdf]

Table S1. Summary of one-way ANOVA results for blood glucose and OGTT outcomes

Outcome	Model	Timepoint / Metric	F-value	p-value
**Blood glucose (BG)**	Normoglycaemic	BG before (baseline)	1.739	0.115
**Blood glucose (BG)**	Normoglycaemic	BG after (day 7)	7.332	<0.0001
**Blood glucose (ΔBG)**	Normoglycaemic	ΔBG (BG after − BG before)	4.351	0.001
**Blood glucose (BG)**	Alloxan-induced diabetic	BG before (baseline)	0.058	1.000
**Blood glucose (BG)**	Alloxan-induced diabetic	BG 0 (48 h post-alloxan)	24.449	<0.0001
**Blood glucose (BG)**	Alloxan-induced diabetic	BG final (day 7)	54.371	<0.0001
**Blood glucose (ΔBG)**	Alloxan-induced diabetic	ΔBG (BG final − BG 0)	28.798	<0.0001
**OGTT**	Normoglycaemic	BG after OGTT	20.613	<0.0001

Abbreviations: BG, blood glucose; ΔBG, change in blood glucose; OGTT, oral glucose tolerance test; NS, not significant.

Note: One-way ANOVA was applied for between-group comparisons; significant omnibus tests were followed by Tukey's HSD ($p < 0.05$). Significant between-group differences are indicated in Tables 2–3 by different lowercase letters, and within-group baseline-to-post differences by an asterisk (*; paired-sample t-test, $p < 0.05$).
